# Exploring the Healing Powers of Histatins: From Oral Health to Therapeutics

**DOI:** 10.3390/ijms26115019

**Published:** 2025-05-23

**Authors:** Saima Usman, Yvonne You, Ahmad Waseem

**Affiliations:** 1 Department of Oral Pathology, National University of Medical Sciences NUMS, Rawalpindi 46000, Pakistan; 2 Centre for Oral Immunobiology and Regenerative Medicine, Institute of Dentistry, Barts and The London School of Medicine and Dentistry, Queen Mary University of London, London E1 2AT, UK

**Keywords:** saliva, antimicrobial peptides, wound healing, histidine-rich basic protein, tissue regeneration, caries prevention, therapeutic peptides, tooth remineralisation, angiogenesis, re-epithelialisation

## Abstract

Histatin peptides are a family of small histidine-rich cationic polypeptides produced by two genes, *HTN1* and *HTN3*. They are found in salivary secretions from the parotid, sublingual, and submandibular salivary glands. These peptides undergo proteolytic cleavages to produce different histatin fragments which play multiple roles including wound healing, maintenance of enamel, and regulation of balance in the oral microbiome. In this review, we explored the expression, structural characteristics, and metal-ion-binding capacities of these peptides and how their functions are modulated by their structure. We also provide here an insight into the potential use of histatins as biomarkers and therapeutic peptides in the management of oral and non-oral diseases including cancer. Potential gaps in the current understanding of histatins that warrant further research have also been highlighted.

## 1. Introduction

Saliva is a biological fluid present in the oral cavity composed of 99.5% water and various components including ions, electrolytes, proteins, and hormones [1]. Ninety percent of all saliva is produced by the major salivary glands including the parotid, submandibular, and sublingual glands. The remaining 10% is contributed by the minor salivary glands which together with the major salivary glands are responsible for the secretion of salivary proteins [2]. Due to the complexity of saliva, it exhibits multiple physiological functions, predominantly underpinned by salivary proteins, including chemical digestion, lubrication, buffering, enamel mineralisation, and protection of the oral cavity [3]. Recent proteomic analysis of saliva has characterised over 3000 distinct salivary proteins and peptides, with 90% being derived from the major salivary glands and resulting from proteolytic degradation [4].

Biomedical researchers today are increasingly focused on developing non-invasive techniques for the diagnosis, monitoring, and treatment of diseases to mitigate the psychosocial trauma and pain that patients often endure during the disease process [5]. Body fluids, particularly saliva, containing a complex mixture of lipids, proteins, small peptides, DNAs, RNAs, and electrolytes have become a key area of interest for non-invasive diagnostic strategies [6]. In this context, antimicrobial peptides, such as histatins found in saliva and ocular fluids, may have broader applications than previously explored [7].

Histatin peptides are endogenous, small, cationic peptides which comprise a family of antimicrobial proteins found in human salivary gland secretions at a concentration of 33.3 +/− 16.7 µg/mL, with histatin-1 contributing the highest concentration [8,9]. The most common histatin molecules found in saliva are histatin-1, -3, and -5, consisting of 38, 32, and 24 amino acids and with molecular weights of 4929, 4063, and 3037 Da, respectively [10]. Each of these peptides share the same first 22 residues with the exception of residue 4 (Glu) and 11 (Arg) in histatin-1. Containing seven histidine residues each, these peptides play a role in wound healing and host defence immunisation of the oral activity due to their antimicrobial properties, as well as remineralisation of the enamel pellicle [11]. Extensive structural and functional studies of these peptides have elucidated specific residues and domains with peculiar activities.

## 2. Gene Expression and Transcriptional Regulation

Amino acid and cDNA sequence analyses, together with evolutionary data, have indicated that histatin peptides are encoded by at least two loci. These have been identified as the *HTN1* and *HTN3* genes mapped to human chromosome 4q13.3 which encode histatin-1 and histatin-3, respectively. Histatin genes have been revealed to arise from the same gene family as statherin proteins from gene-duplication events [12]. *HTN1* and *HTN3* both comprise six exons. Whilst all of the six exons in *HTN1* are protein-coding, only exons 2–5 are translated in *HTN3.* These protein-coding exons are alternatively spliced and ligated to give rise to histatin-2, -4, -6, and -7–12 [13]. The transcriptional regulation of these genes remains elusive. However, a HTN27 box located approximately 2.3 kb upstream of the first exon of *HTN1* has been shown to strongly stimulate *HTN1* transcription in human salivary gland cells [14].

## 3. Post-Translational Modifications

The absence of histatin-2 and -4 in freshly collected saliva from the parotid gland and their later appearance following autoproteolytic degradation of the major histatin peptides indicates that these peptides undergo an intricate proteolytic processing [15]. Studies monitoring the formation and degradation of histatin fragments in whole saliva identified the sites first targeted for cleavage in histatin-1, -3, and -5, suggesting that proteolytic enzymes have a greater affinity for these sites [16]. Reverse-phase high-performance liquid chromatography and electrospray ionisation mass spectrometry techniques have confirmed that histatin-3 is the first histatin peptide to undergo proteolytic cleavage to generate histatin-5 and -6 which share sequence similarity except for the presence of an additional C-terminal arginine residue [17,18]. It has been suggested that histatin-6 is the first fragment produced from proteolytic cleavage based on tandem mass spectrometry studies on human saliva. As the only C-terminal fragments identified corresponded to residues 26–32, 28–32, and 29–32 of histatin-3, it is suggested that the initial cleavage occurring at residue 25 corresponds to an arginine residue [19]. Human salivary proteases are also shown to target the arginine residue at position 22 and tyrosine residue at position 24 during primary cleavage in histatin-3 [16]. Although cleavages at the lysine residues at position 13 and 17 in the C-terminal region are observed in all of the three major histatin peptides and are primary targets in histatin-1 and -5, these cleavages occur after those seen at position 22 and 24 in histatin-3, suggesting that salivary proteases display higher affinity for these residues [16]. As the functional activities of histatins, such as their antifungal, wound-healing, and metal-binding abilities, are mostly unaffected by these primary cleavages, it indicates that these peptides maintain sustained functionality within the proteolytic oral environment [16].

Post-translational processing of histatin-3 is thought to give rise to histatin-4 and histatins-7–12, as these peptides consist of residue sequences identical to those seen in histatin-3 [7]. For instance, the amino acid sequence of histatin-4 is identical to the last 20 residues of the C-terminal end of histatin-3 and is suggested to arise from a trypsin-like cleavage of histatin-3 between lysine and arginine residues 11 and 12 [7,13]. Histatin-7 and -8 which show sequence similarity to residues 12–24 and 13–24 of histatin-3 may be generated by proteolytic cleavages at lysine and arginine 11 and 12, respectively, of histatin-3 or histatin-5 with an additional cleavage at tyrosine 24 if generated solely from histatin-3 [13]. Histatin-9 and -10 arise from cleavages at lysine 11 and arginine 12 and 25 of histatin-3, resulting in fragments with sequence similarity to residues 12–25 and 13–25, respectively. Alternatively, they may arise from cleavage of the same residues excluding the cleavage at position 24 from histatin-6 [13]. Cleavages at residues 4, 11, and 12, corresponding to alanine, lysine, and arginine, respectively, give rise to histatin-11, which is identical in sequence to residues 5–11, and histatin-12, which shares a sequence of residues 5–12 [13].

Post-translational modification of histatin-1 includes the phosphorylation of serine at position 2 which is not present in histatin-3 and -5, suggesting that the salivary gland kinase is specific for histatin-1 [19]. Additionally, some histatin-1 derivatives found in submandibular and sublingual gland secretions contain sulphated tyrosine residues [20]. Histatin-2 has been reported as a degradable product and non-phosphorylated form of histatin-1 as it shares the same C-terminal 26 residues but lacks the phosphorylated serine residue at position 2. This peptide is thought to be generated by a trypsin-like cleavage of histatin-1 between arginine residues 11 and 12 [13]. Understanding the proteolysis of histatin peptides is crucial for elucidating their biological activities.

## 4. Structure and Functional Domains of Histatins

There is little available literature on the secondary structure of histatin peptides; however, circular dichroism and nuclear magnetic resonance studies have revealed that histatins adopt different conformations in different solvents [21,22]. The structural and chemical configuration of histatins is linked to its ability to bind to multiple different ligands including metal ions [23,24] and other molecules such as sigma-2 receptors [25]. In aqueous solution, histatins display weak amphipathic character [12]. Histatin-5, in particular, adopts a random coil conformation in aqueous solutions and a largely α-helical conformation in non-aqueous solvents including methanol [26], dimethyl sulfoxide [27], and trifluoroethanol [22], suggesting that histatins adopt a helical conformation in a hydrophobic environment [21,26]. Whilst helical structures are normally stabilised by side-chain hydrogen bonding and salt-bridge interactions in aqueous solution, this was not observed in histatin-5 [27]. Histatin-3, on the other hand, showed a lower tendency to adopt a helical structure in aqueous solution, and in a 50:50 mixture of water and dimethyl sulfoxide solution, it became more ordered [21].

Out of the 24 amino acids in histatin-5, 13 can act as potential ligands for metal coordination, leading to the formation of stable conformational complexes and bonds and potential differences in their interactions with macromolecules. Structural and functional characterisation of histatin-5 using two-dimensional proton NMR spectroscopy showed that the binding of metal ions to the polypeptide leads to stabilisation of the helical conformation in a solution of trifluoroethanol and water [28]. The N-terminal of histatin-5 contains a Cu^2+^/Ni^2+^-binding motif, termed the ATCUN motif, which consists of a histidine residue at position 3 (XXH, where X represents any residue), and a Zn2+-binding motif (HEXXH, where X represents any residue) [29]. The ATCUN motif adopts a square planar geometry, and upon metal ion binding, it has been shown to induce the production of reactive oxygen species (ROS) in the presence of ascorbic acid or magnesium monoperoxyphthalate, which may be potentiated in the antifungal activity of histatins [23].

The HEXXH motif is a common zinc-binding motif found in many proteins. Corresponding to residues 15–19 in histatin-1, -3, and -5, it adopts a helicoidal conformation and has been shown to preferentially bind zinc ions in histatin-5, enabling the peptide to fuse with negatively charged vesicles [28,29]. This causes histatin-5 to undergo a conformational change which stabilises its α-helical structure, as revealed by circular dichroism spectroscopy [28]. As the HEXXH motif is found twice in histatin-1 and once in histatin-3, it is likely that the binding of zinc ions to these peptides exhibits similar properties to those seen in histatin-5. An amyloid peptide containing an identical zinc-binding motif to that present in histatin peptides was found to also stabilise α-helical conformation in solvents [30]. NMR spectroscopy studies of P-113, a 12-amino-acid-long peptide spanning residues 4–16 of histatin-5, identified that zinc ions are coordinated to the HEXXH motif by nitrogen donor atoms of alanine (position 1) and histidine (position 4, 5, and 12) residues [31]. From this, it is possible to conclude that zinc ions are similarly coordinated in full-length histatin peptides. Whilst zinc binding is preferential, cobalt ions are also able to bind to the first HEXXH motif in histatin-5 via two histidine Nε atoms and one Nδ atom and two histidine residues in the second HEXXH motif via one Nε atom and one Nδ atom [32]. It has been proposed that the imidazole ring in the histidine residue and the carboxylic acid group of glutamic acid in the HEXXH motif may also stabilise hydrogen bonding with a water molecule [29]. With this, it is possible to theorise that metal coordination may confer an optimal structural configuration to enable histatin peptides to interact with other peptide molecules.

## 5. Domain-Specific Functions

### 5.1. Antifungal Properties

The warm, moist environment of the oral cavity is conducive to the growth of multiple microorganisms and requires complex defences to prevent infection. Histatins help to exhibit a broad spectrum of antifungal activity against fungal oral pathogens including *Candida albicans* (*C. albicans*), *Cryptococcus neoformans*, and *Aspergillus fumigates*, with *C. albicans* being responsible for the most common oral fungal infection, candidiasis [33].

Histatin-1, -3, and -5 all exhibit antifungal activity against *C. albicans*; however, histatin-5 has been shown to display the most efficient and strongest level of antifungal activity by killing both blastopore and germinated forms [9,10,26]. Solid-phase procedures identified that residues 9–24 of histatin-5 had higher levels of antifungal activity compared to residues 1–16, indicating that antifungal activity is attributed to the C-terminal end of the peptide. Increasing the length of the C-terminal peptide from 10 residues to 16 residues displayed an increase in antifungal activity of the peptide with an increase of approximately 40–50% from 0% by lengthening the peptide from 10 to 12 residues at a concentration of 25 µM. Whilst the C-terminal fragments 11–24 and 9–24 displayed 80 and 90% antifungal activity, respectively, compared to the full-length peptide construct, the shorter fragments were far less active. Thus, increasing the chain length of the C-terminal sequence from 12 to 16 residues enhanced the antifungal activity of histatin-5, suggesting that a minimum peptide length of 12 residues is required for optimal biological activity [26].

**Figure 2:**
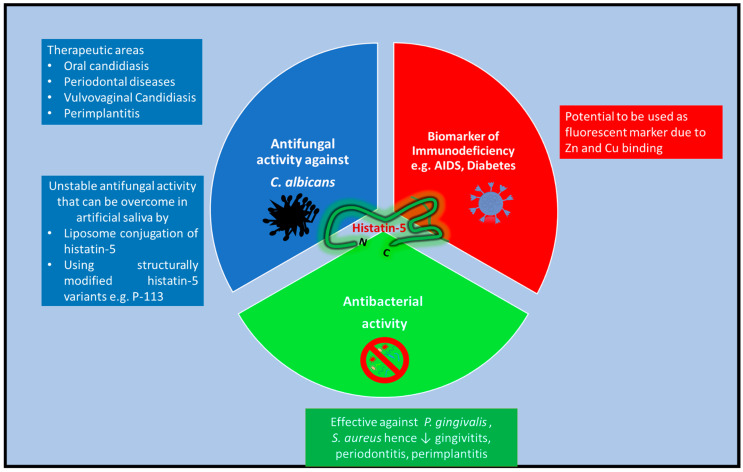
Histatin-5’s applications as a biomarker and therapeutic peptide. Histatin-5 therapeutic peptides can be modified to overcome unstable activity.

Several mechanisms of histatin-induced candidacidal activity have been proposed including differences in the mode of interaction with the fungal cell wall and cell membrane, as well as the ultimate cellular target of these peptides. One such mechanism of cellular uptake is the ability of histatins to bind to and integrate with the negatively charged lipid bilayer of the fungal cell membrane, resulting in the formation of transmembrane pores. This leads to microleakage of the fungal cell contents including potassium ions and ATP, resulting in osmotic imbalance and ultimately microbial cell death [9,34,35]. It has also been proposed that histatins undergo a conformation change from a random coil in aqueous solution to an α-helical conformation when in close proximity to the fungal cell membrane, suggesting that antifungal activity may rely on the structural conformation of the peptide [26,36]. In contrast, replacement of specific residues in histatin-5 with proline to prevent alpha-helix formation and insertion into the fungal cell membrane did not reduce the efficacy of its antifungal activity, indicating that the fungicidal mechanism of histatin-5 is not reliant on its insertion into the fungal cell membrane [37]. Histatins are also suggested to target the mitochondria of respiring fungal cells based on early colocalisation analysis studies where they form ROS which inhibit mitochondrial respiration leading to cell death [38,39,40,41]. Mass spectroscopy studies have indicated that the binding of copper ions to the ATCUN motif in histatin-5 is required for the production of ROS following its cellular uptake [23]. This candidacidal mechanism is supported by studies using histatin-5, indicating that fungal cells subject to treatment with energy inhibitors or mutation of the mitochondrial DNA resulted in a reduced susceptibility to this peptide [42]. However, it has also been suggested that ROS play no role in the antifungal activity of histatin-5 as the application of an ROS scavenger elicited no inhibitory effects on the killing of *C. albicans* cells [43]. In vivo fungicidal assays of *S. cerevisiae* demonstrated that the antifungal mechanism of histatin-5 involves binding to the heat shock protein, Ssa1/2, which are envelope binding receptors on the cell wall of *C. albicans* cells [34,44]. The uptake of histatin-5 following cell wall binding is proposed to utilise the cell wall polyamine transporters Dur3 and Dur31 of *C. albicans* in an energy-dependent process [45]. Studies also suggest the possibility that histatin-5 is localised into the vacuoles of fungal cells via an endocytic pathway, as endocytic mutant *C. albicans* cells displayed reduced localisation; however, this is not significant for the antifungicidal activity of histatin-5 [46,47]. The ability of histatin-5 (Figure 2) to show fungistatic and fungicidal activities against strains resistant to pore-forming antifungal azole and amphotericin drugs in vitro including *C. galbrata* and *C. krusei* suggests that histatins may be utilised as an alternative antifungal therapy against antifungal-sensitive and antifungal-resistant strains of these microorganisms [48]. Yeast two-hybrid analysis also highlights interactions between Ssa1/2 cell surface receptors and histatin-3, suggesting that histatin-3 also exhibits a similar antifungal mechanism to histatin-5 [34].

P-113 (from residue 4–15 of histatin-5; a 12-residue peptide) was identified as the shortest fragment of histatin-5 that can retain the antifungal activity of its full-length parent peptide, using in vitro killing assays against *C. albicans* and other *Candida* species [49]. The substitution of two adjacent histidine residues (H^7^H^8^) in the full-length histatin-5 without subsequent change in the proteins’ structural conformation resulted in a 8–20-fold reduction in its antifungal activity, suggesting that histidine residues are necessary for the candidacidal activity of histatin-5 [48]. On the contrary, individual replacement of the histidine residues at positions 4, 5, and 12 of P-113 with other hydrophobic residues did not affect the anticandidal activity of this peptide, suggesting that histidine is not an essential residue for eliciting antifungal activity in vitro [49]. The transport of P-113 into the cytosol of *C. albicans* cells has been shown to rely on the cationic lysine residues at position 2 and 10 [50]. Further research is required to determine if histidine residues play a role in the mechanism of action of P-113 in vivo, including its stability and ability for tissue binding.

### 5.2. Antibacterial Properties

Although some studies suggest that histatins exhibit limited or no antibacterial effects against common oral bacteria such as *Streptococcus mutans* (*S. mutans*) [51], others show that the cationic properties of histatin promote bactericidal effects [52,53,54,55,56]. The negative charge on bacterial cell membranes is conferred by the large proportion of acidic phospholipids. As histatin peptides are positively charged, they are thought to undergo electrostatic interactions with target bacterial cell membranes and subsequently integrate into the lipid bilayer. This ionic interaction leads to thinning of the cell membrane which is an essential step for their antibacterial activity [57]. *S. mutans* is a Gram-positive facultatively anaerobic bacterium and a major aetiological agent of tooth decay, as it forms biofilms on the enamel surface. The interactions of histatin-1 and another salivary protein, statherin, with the enamel pellicle competitively inhibit the adsorption of adhesion-promoting high-molecular-weight glycoproteins (HMWGPs) on the hydroxyapatite surface; these HMWGPs facilitate the attachment of *S. mutans* onto the enamel surface [58]. This antibacterial property of histatin-1 can be attributed to the negative charges present at the N-terminal of the peptide, as removal of the negative charges diminished its inhibitory effects [58].

Periodontal disease is characterised by an increased level of inflammatory exudate consisting of inflammatory mediators and tissue-breakdown products within the periodontal pockets, owing to the presence of pathological bacteria. *Porphyromonas gingivalis (P. gingivalis)* is a Gram-negative bacterium responsible for the development of periodontal inflammation and peri-implantitis. It has been revealed that histatin-5 can inhibit the production of inflammatory cytokines by this bacterium in human gingival fibroblasts by altering its membrane function and metabolic processes and prevent its trypsin-like activity on the periodontal tissues [59,60].

### 5.3. Enamel Fortification

The acquired enamel pellicle (AEP) is a thin biofilm covering the oral mucosa and tooth surfaces. This layer is formed by the adsorption of organic and inorganic molecules from saliva onto the enamel surface. The main function of AEP is the lubrication and protection of teeth from demineralisation; in addition, it also helps in the remineralisation process. The AEP also provides adhesion sites for polymicrobial colonisation during biofilm (plaque) formation [61].

Salivary peptides, especially histatins, are among the first peptides to be adsorbed on the hydroxyapatite of enamel during AEP formation [62]. Histatins are multifunctional molecules possessing antibacterial, antiviral [63], and antifungal properties [10,38,53]. As discussed before [64], these peptides also inhibit adsorption of high-molecular-weight glycoproteins on the tooth surface that provide adhesion sites for cariogenic bacteria. In addition, these peptides also prevent crystal growth of calcium and phosphate salts in saliva, thus maintaining high calcium and phosphate ionic levels. Collectively, these features contribute to the maintenance of enamel integrity [65].

Although, normally, histatins are highly sensitive to proteolytic degradation in whole saliva, binding to the AEP has been shown to exert a protective effect against further proteolysis, possibly by preventing access of the proteases to their preferred cleavage sites, and also by blocking precipitation of calcium and phosphate on the enamel surface [66,67]. Several studies have shown that phosphorylated forms of histatins are more potent in protecting enamel from demineralisation. It has been reported that the phosphoserine at position 2 in histatin-1 or synthetically introduced into histatin-3 conferred a significantly higher degree of hydroxyapatite adsorption and protection of the enamel against demineralisation compared to unphosphorylated histatins [66,68]. This is consistent with previous studies indicating that adsorption to hydroxyapatite is greater in full-length histatin-1 compared to recombinant histatin-1, lacking the phosphate at position 2 [69]. In the absence of histatins, however, no reduction in demineralisation was reported, indicating that phosphoserine is not the only determining factor affecting hydroxyapatite adsorption [70].

Adsorption to hydroxyapatite is an important feature of histatins, and, in fact, histatin-1 was first identified when it was found to adsorb to hydroxyapatite powders [71]. The relatively lower affinity of histatin-3 and -5 to hydroxyapatite is proposed to be due to the presence of an SXA motif (where X presents any residue) instead of the SXE motif, which is present in histatin-1 [72]. The SXE motif can be phosphorylated and is considered to be responsible for the binding of calcium ions [72]. Despite this, in vivo studies have identified full-length histatin-3 and -5 peptides present within the AEP, suggesting that upon binding to hydroxyapatite, these peptides are able to resist proteolysis, possibly by adopting a favourable conformation that resists trypsin-like protease activity [66]. The identification of histatin-1 fragments in the human AEP in vivo may suggest that these peptides may still exert protective properties following proteolytic cleavage [73,74].

The adsorption of histatins to the AEP in the presence of other proteins, however, has been shown to influence the adsorption behaviour of these molecules; thus, further studies are required to elucidate the functional effects of histatins in multi-protein systems [68]. Moreover, the precise mechanism by which histatins reduce demineralisation needs to be explored further.

### 5.4. Immunomodulation

Periodontal bacteria can produce lipopolysaccharides, which trigger the activation of immune signalling cascades, leading to periodontal destruction [75]. Histatins have been revealed to display immunomodulatory and anti-inflammatory effects, thus protecting the periodontium. In human oral fibroblasts, histatin-3 binds to the heat shock cognate protein, HSC70, at its substrate-binding domain (residues 385–543) and inhibits the activation of toll-like receptor signalling pathways and subsequent inflammatory cytokine production [76,77]. Histatin-1 has been shown to limit the inflammatory response in a different way by reducing the production of nitric oxide triggered by lipopolysaccharides, inflammatory cytokines, and other inflammatory mediators that participate in the c-Jun N-terminal kinase (JNK), MAPK, and NF-kB inflammatory signalling pathways in macrophages [78]. Histatin-5 also showed an inhibition of periodontal inflammation and alveolar bone resorption in rats with experimental periodontitis; therefore, it is plausible that histatin-5 regulates periodontitis in a similar manner in humans [60].

A hallmark of gingival and periodontal disease is an increase in both host and bacterial proteolytic enzymes, including matrix metalloproteinases (MMPs), which play a role in the destruction of periodontal tissues. Experiments comparing the activity of various histatin-5 fragments on host-derived MMP-2 and -9 concluded that residues 9–22 showed identical inhibitory effects on MMPs as those of the full peptide, suggesting that these residues comprise an antibacterial C-terminal functional domain [12,41]. Histatin-5 is also able to deprive microorganisms of the copper and zinc ions necessary for enzyme function and microbial growth by sequestering and binding to these ions via specific binding sites [12,79]. The low dissociation constants of histatin-5 with copper and zinc ions suggest that the metal-binding motifs of the peptide can bind these ions in saliva under physiological conditions [23,41]. It is suggested that histatin-5 can also bind these ions present within the active domain of MMPs, resulting in their inhibition. It has been demonstrated that histatin-5 is also capable of competitively and non-competitively inhibiting arginine- and lysine-specific gingipains, which constitute a class of enzymes involved at the onset of periodontitis produced by *P. gingivalis* [41]. The topical application of histatin-5 and its fragment P-113 to beagle dogs with gingivitis was shown to significantly prevent plaque formation, bleeding on probing, and the onset of gingival inflammation [80].

### 5.5. Wound Healing

Wound healing is a vital biological process that restores tissue integrity after injury. This process involves four coordinated phases: haemostasis (blood clotting), inflammation, proliferation (characterised by new tissue formation through collagen synthesis and angiogenesis), and remodelling [81]. Wound healing in the oral cavity is notably faster and more efficient than in the skin, partly due to the presence of histatin peptides [82,83]. Histatins can influence various stages of wound healing, including cell migration, spreading, adhesion [84,85], angiogenesis [86], and the suppression of inflammation [59,77,78,87,88].

Notably, histatin-1 and -3 actively promote epithelial migration, unlike histatin-5 [89]; this is different from classical mitogenic factors such as epidermal growth factor (EGF) [89,90]. One study has described the possibility of histatin-induced cell migration via G-protein-coupled receptors (GPCRs), as the pertussis toxin inhibited histatin-stimulated keratinocyte motility [91]. The influence of histatin extends beyond epithelial cells, facilitating migration in multiple cell types, including fibroblasts [91,92], osteoblasts [93], adipocytes [94], and endothelial cells [86,92]. The situation becomes more complicated by the results indicating that histatins can induce cell migration in different cells, but the mechanisms are variable and cell-specific; for example, in fibroblasts, the mammalian target of rapamycin (mTOR) signalling pathway has been suggested to be involved [95], whereas in endothelial cells, the ERK1/2 signalling pathway may be involved [86], similar to epithelial cells [89].

In addition to migration, histatins also support cell spreading and extracellular matrix (ECM) attachment, a crucial step during wound healing and tissue regeneration. Studies have used retinal epithelial cells, colorectal adenocarcinoma cells, and fibroblasts [84], as well as endothelial cells, on ECM matrices [85,86].

Angiogenesis is a vital process in wound healing, where the stimulation and proliferation of endothelial cells lead to the formation of new blood vessels, ensuring an adequate nutrient supply for tissue regeneration [96]. In endothelial cells, migration and angiogenesis induced by histatins involve VEGFR2 signalling [86,97]. The RIN2/Rab5/Rac1 axis, critical for vascular morphogenesis [98], regulates histatin-1’s effect on endothelial cell spreading and barrier integrity [86]. In vivo studies also confirmed that histatins promote wound healing through angiogenesis, endothelial cell adhesion, and barrier integrity [85,86,99]. In a wound-healing mouse model, topical application of histatin-1 (10 µM) demonstrated significantly improved acute wound healing compared to an acellular dermal matrix paste [100].

Despite significant progress, histatin internalisation and trafficking remain insufficiently understood, warranting further investigation. They are suggested to be stereospecific membrane receptor-mediated and energy-dependent (via GPCR/endocytosis/ERK signalling process) [89,91], targeting the mitochondria, endoplasmic reticulum [91,101], and endosomes (in endothelial cells) [86], thereby increasing metabolic activity and cell activation. An endoplasmic reticular protein, TMEM97/sigma-2 receptor, involved in cholesterol processing, cell migration, neurodegenerative diseases, and cancer has been shown to be the downstream target (receptor) of histatin-1 (ligand) in epithelial cells [25].

The wound-healing properties observed in histatin-1, -2, and -3 are thought to be attributed to their C-terminals. This is supported by in vitro studies demonstrating that histatin-5, which is derived from the N-terminal 24 residues of histatin-3, does not display any wound-closure activity compared to histatin-1 and -3 [16]. In vivo scratch assays of human corneal limbal epithelial cells and serial truncation experiments on the efficacy of histatin-1, after progressively deleting residues, identified that residues 20–32 (SHREFPFYGDYGS) formed the minimal active wound-healing domain of histatin-3. Histatin-5, on the other hand, was shown to only retain the SHR portion of the histatin-1 wound-healing domain [102]. Similar scratch assays performed in vitro identified that a 5-amino-acid-long C-terminus domain (SHRGY) of histatin-5 is required to promote epithelial cell migration during wound closure, as constructs without this sequence showed no significant changes to the rate of wound closure [102]. It has been demonstrated that cyclisation of histatin-1 increased its wound-closure activity by approximately 1000-fold, revealing that recognition and binding of histatin-1 to its cognate receptor requires the adoption of a specific spatial conformation [89,103].

### 5.6. Possible Role in Cancer

**Figure 1:**
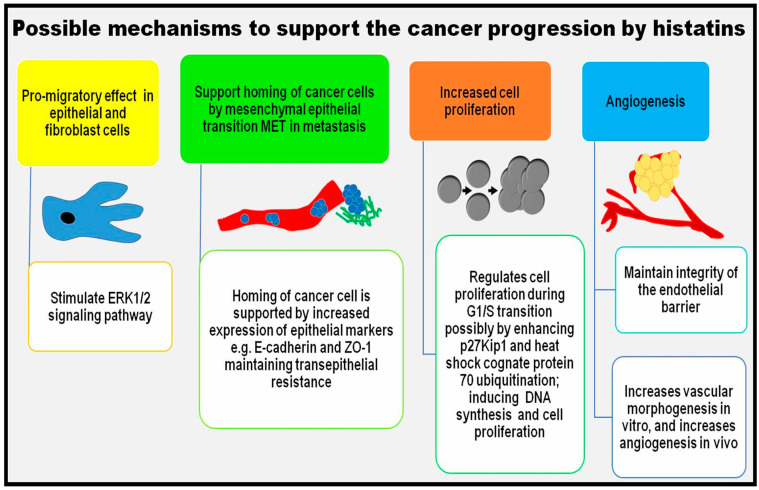
Possible/speculated mechanisms through which histatins, especially histatin-1 and -3, can support cancer progression.

The promigratory and pro-angiogenetic roles of histatins can be potentially linked to cancer development and progression, as previous studies have demonstrated the increased migration rates induced by histatins on epithelial tumour cells, including MCF-7 breast carcinoma [92], Caco-2 colorectal carcinoma, and TR146 oral cancer cells [84,85,86]. Histatin-3 appears to regulate G1/S transition in oral cells through its association with HSC70 and p27 (Kip1) [104]. Also, a high-throughput saliva proteomic analysis of HNSCC samples showed that HTN3 fragments were highly expressed, indicating their involvement in OSCC progression [105]. Furthermore, in a gene profile analysis of HNSCC tissue samples, *HTN1* was one of the top fifty dysregulated genes [106]. In addition, the gene expression signature of oral squamous cell carcinoma samples based on a GeneChips array study suggested that *HTN1* and *HTN3* were highly expressed in advanced-stage HNSCC [107,108]. Histatin-1 can enhance cell–cell adhesion markers like E-cadherin and ZO-1 in Caco-2 cells while counteracting the effects of EMT-inducing agents such as EGF and TGF-β. These findings were observed in spheroid assays using TR146 epithelial cells [85]. In addition, immunohistochemistry analysis of 98 samples of head and neck squamous cell carcinoma (HNSCC) showed higher expression of histatin-1, and a positive correlation between PD-L1 and histatin-1 was associated with the progression of HNSCC [109]. Based upon the available literature, it can be speculated that although histatins appear to be involved in cancer progression, the precise gain or loss of functional roles remain unclear and unexplored, particularly in oral malignancies. Future research is necessary to find out the precise role of histatins in cancer progression and uncover their broader potential in cancer biology. All possible/potential mechanisms of cancer progression that can be influenced by histatins have been summarised in Figure 1.

## 6. Biomedical Applications of Histatins

### 6.1. Histatins as Biomarkers

A biomarker, also known as a molecular marker or signature molecule, is a biological molecule present in blood, other bodily fluids, or tissues. It serves as an indicator of a normal or abnormal process, condition, or disease. Biomarkers can also be used to assess how effectively the body responds to a treatment for a specific condition or illness (https://www.cancer.gov/publications/dictionaries/cancer-terms/def/biomarker; accessed on 20 April 2025). In addition to being recognised as antimicrobial peptides, histatin salivary levels also correlate with different physiological and pathological disease processes. This makes them valuable as diagnostic and disease-monitoring potential biomarkers, as outlined in Table 1. Their zinc and copper ion-binding domains can potentially be conjugated to metal ions with fluorescence or magnetic properties to be utilised as fluorescent biomarkers for diagnostic and monitoring purposes. However, this capability has yet to be utilised in clinical applications.

### 6.2. Histatins as Therapeutic Peptides

#### 6.2.1. Antimicrobial Therapy

Histatin peptides, being antimicrobial, can be used in oral as well as non-oral disease management as an alternative to conventional drugs. For therapeutic applications, these are especially recommended where the infection is localised and accessible via topical delivery, such as for the treatment of candidiasis (thrush) and mucositis in the oral cavity. Non-oral diseases include resistant skin infections and lung infections [123,124].

The antimicrobial potential of histatins can also be exploited in artificial salivary products for the management of patients with salivary dysfunction or xerostomia. The potential for histatins to be used in artificial saliva substitutions in patients with salivary gland dysfunction is indicated by the increased incidence of oral fungal infection in the absence of histatin molecules. Moreover, their antifungal properties suggest their effectiveness as topical histatin preparations and histatin-containing denture base acrylics to prevent *C. albicans* infection. Further research is, however, necessary to determine optimal expression systems for the construction and later purification of histatin variants with enhanced antimicrobial properties, which can be used to deepen our understanding of their functional mechanisms and aid the manufacture of novel histatin-based therapeutic agents on a grander scale. There is one putative active domain in histatin-5 called Dh5 (residues 11–24) that has been used as a scaffold in the design of new peptides when looking for new medicines [125]. The therapeutic applications of histatin-5 are summarised in Figure 2.

#### 6.2.2. Caries Prevention Therapy

**Figure 3:**
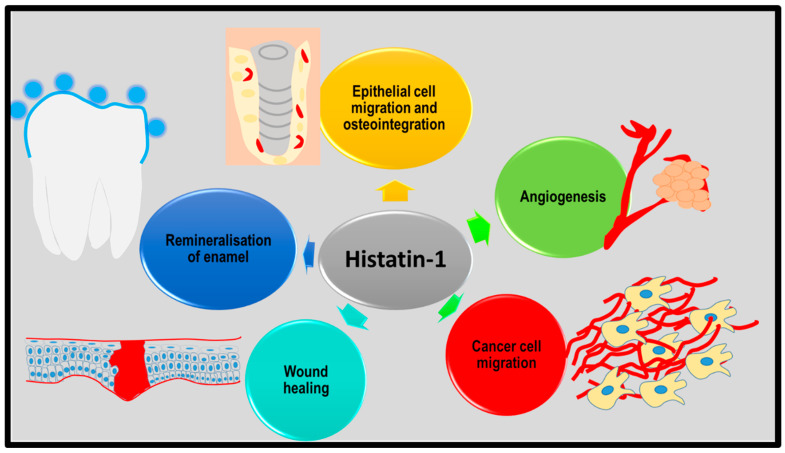
Biomedical applications of histatin-1. Histatin-1 can be used in caries prevention as it remineralises enamel and enables tissue regeneration and implant osteointegration. Histatin-1 also supports cancer progression, especially in head and neck cancer; however, the precise mechanism is inexplicit and warrants further research.

As histatins, especially histatin-1 (Figure 3), help with remineralisation and restrict microbial biofilm formation on teeth, histatin-based therapeutic products such as gels, toothpastes, and varnishes can be synthesised for caries prevention.

#### 6.2.3. Tissue Bioengineering

Tissue engineering is an emerging field, and histatins are attractive components for tissue regeneration due to their antimicrobial actions [10,126], supportive role in angiogenesis [86], involvement in epithelial cell migration [89,91], and anti-inflammatory potential [77,78]. In vivo studies involving rodent burn models [127] and skin wound models in diabetic rats [128,129] have confirmed histatins’ potential applications in tissue regeneration and wound healing. The antimicrobial properties of histatins can be exploited in the synthesis of novel therapeutics that can replace conventional antibiotics to overcome wound infections and antibiotic resistance. For example, the recent development of an antibacterial, self-healing adhesive nanocomposite hydrogel possessing ideal mechanical and biological properties was shown to promote skin full-thickness wound regeneration in mouse models [130,131]. These hydrogels, which significantly promote wound closure, collagen deposition, and angiogenesis, may be modified to efficiently release histatin peptides to accelerate wound healing, including those infected by bacteria.

Histatins exhibit remarkable potential in bone repair and regenerative medicine by activating pre-osteoblasts and inducing the expression of key osteogenic markers, including osteocalcin, osteopontin, and Runx2, while also increasing alkaline phosphatase (ALP) expression and enzymatic activity [121,132]. Additionally, histatin-1 supports tissue regeneration by enhancing surface adhesion and migration in non-osteogenic cells, such as primary mesenchymal cells from dental pulp and tooth apical papilla [121].

Histatin-1 has also been found to improve the efficiency of bone morphogenetic protein-2 (BMP-2) in stimulating ectopic bone formation in vivo, although the underlying mechanisms remain unexplored [133]. Its regenerative potential has been validated across several experimental models, including an orthotopic bone-healing model [134], a monosodium iodoacetate (MIA)-induced osteoarthritis rat model [135], and a model of bisphosphonate-related osteonecrosis of the jaw (BRONJ), where it has been shown to counteract the cytotoxicity induced by zoledronic acid (a bisphosphonate) on pre-osteoblasts and endothelial cells [136].

Advancements in bioengineering suggest that conjugation of histatins with biocompatible materials may further enhance their therapeutic potential. For instance, histatin-5 conjugated with a titanium-binding peptide effectively prevents *P. gingivalis* adherence and biofilm formation on titanium implants, thereby mitigating peri-implantitis and improving osteointegration [137]. These insights underscore the promising applications of histatin-1 in osteogenic differentiation and functional tissue regeneration, paving the way for novel strategies in bone and pulpal regenerative medicine. Future investigations could focus on elucidating the molecular mechanisms governing histatin-mediated bone regeneration and optimising its integration with biomaterial scaffolds for next-generation regenerative therapies.

#### 6.2.4. Anticancer Therapy

Histatins have emerged as promising anticancer peptides, with studies demonstrating their potential to enhance the efficacy of traditional chemotherapeutic agents. Specifically, histatin-1 has been shown to increase the sensitivity of HNSCC cells to cisplatin, allowing for a reduction in the required drug concentration while maintaining therapeutic effectiveness [138].

Beyond their role in chemotherapy efficacy, histatins exhibit nuclease-like activity and possess metal-binding sites, positioning them as attractive candidates for artificial metalloscissors in cancer therapy [12]. Metal complexes, a key category of artificial metalloscissors, can facilitate nucleic acid strand cleavage, offering a targeted approach to disrupting disease-related DNA/RNA. Unlike conventional drugs, these metalloscissors act without enzyme-like functions, providing a distinct mechanism for oncological and antimicrobial interventions. This concept draws inspiration from natural metallonucleases and metallopeptide antibiotics [139].

By leveraging their intrinsic nuclease activity and metal-binding properties, histatins hold potential as therapeutic peptides capable of executing nucleic acid modifications for cancer treatment. Further investigation into their molecular mechanisms and integration with bioengineered drug delivery systems may unlock new avenues for precision oncology.

## 7. Overcoming Limitations in Therapeutic Applications of Histatins

### 7.1. Combined Histatin Preparations for Enhanced Functionality

Varying concentrations of different histatin peptides may facilitate the differentiation of diseased and healthy states without surgical intervention; therefore, different histatin peptides can be combined together to enhance their therapeutic efficiency. Their natural presence in human saliva and lack of known cross-reactivity with host tissues render these peptides advantageous for use in the oral cavity as potential therapeutic agents compared to conventional therapies. Clinical studies have demonstrated that modified histatin preparations show high biocompatibility in the oral cavity and can slow down plaque formation, thereby reducing the severity of oral diseases [38].

### 7.2. Overcoming Proteolytic Instability

The therapeutic potential of histatins against fungal infections is restricted by their instability against proteolysis [16]. For instance, a study demonstrated that histatin-5 is cleaved and inactivated by secretory aspartic proteases (Saps) produced by *C. albicans*. The same study also identified that a single-residue substitution, K17R, in histatin-5 confers increased resistance to proteolysis by Saps, whilst K11R substitution enhanced its antifungal activity [140]. Thus, structural and functional analysis of histatins is crucial for identifying their proteolytic susceptibility and will impact their use as therapeutic agents. Modification of histatins’ structures, including shortening the peptide or substituting amino acids, may confer resistance to proteolytic degradation whilst retaining their activity and can help to reduce the cost of their manufacture and production [64,140,141].

### 7.3. Achieving Gradual and Constant Release of Histatins

One of the major drawbacks in using histatins as therapeutic peptides is that they cannot be constantly and gradually released. Histatins can form complexes with other proteins, rendering them non-functional; for example, histatin-5 is shown to form a complex with amylase in saliva that does not display antifungal activity [142]. Thus, developing a mode of gradual and constant release of histatins is needed in the treatment of oral and non-oral lesions.

### 7.4. Improving Histatin Delivery in Therapeutics

Histatins as therapeutic peptides can be combined or incorporated into different vehicles for improved delivery and bioavailability. Unremitting identification of a suitable and efficient drug delivery system which supports stabilisation of histatins is fundamental for their promotion as a therapeutic agent. Histatins can be engineered into nanocarriers, e.g., liposomes or micelles, to improve drug stability and targeted release in oral tissues [143]. The use of liposomes as carriers for histatins has many advantages because of their high biocompatibility, attributed to their phospholipid membranes, and their ability to protect their contents from proteolytic degradation [144]. Based on this, synthetic histatin-5 peptides have been synthesised using solid-phase synthesis techniques and purification and incorporated into liposomes using a thin-film hydration technique. It was observed that these liposomes were able to gradually release histatin-5 over a period of 96 h and control yeast growth for 72 h, thus promoting its availability at the site of action and prolonging its antifungal effects [145].

### 7.5. Modifying Peptide Length to Ensure Best Possible Drug Efficiency

Histatin bioengineered therapeutic peptides of varying lengths can be made while keeping their functionality to improve their therapeutic efficiency. Fragments derived from full-length histatin-3 and -5 peptides display the same or similar levels of antifungal activity, possibly highlighting their use as short-term antifungal peptide drug therapies. Animal studies and human clinical trials showed that P-113, the 12-residue-long amidated fragment of histatin-5, has the potential to prevent the development of gingivitis, with no side effects. It shows its potential use as a safe daily mouth rinse, as demonstrated in a study which revealed that a dose level of 0.01% of this peptide was effective in significantly preventing the development of plaque and gingivitis in an experimental human model of periodontal disease [146]. The lack of mucosal irritation, staining, and other notable side effects of common daily mouth rinses highlights the potential of P-113 to be used as a therapeutic product against periodontal disease [146]. The potential for histatins in the treatment of vulvovaginal candidiasis in mouse models has been highlighted by the design of small antifungal peptides inspired by the structure of histatin-5 which show a selective preference for fungal cells over bacteria and mammalian cells and promote fungal cell death by targeting the nucleus and mitochondria [147].

Bioengineered peptides synthesised to enhance the activity and bioavailability of histatin therapeutic peptides are summarised in Table 2. Antimicrobial peptide modifications intended to improve their activity against a pathogen may have unpredictable and undesirable side effects on other pathogens, as summarised in Table 3.

## 8. Conclusions

Histatins, a remarkable group of histidine-rich cationic peptides, hold immense promise in biomedical applications. Their ability to prevent enamel demineralisation, accelerate wound healing, and combat *C. albicans* and bacterial infections underscores their potential as transformative therapeutic agents. As research continues to unravel their precise 3D structure and functional mechanisms, the possibilities for innovation expand. Future advancements in bioengineering could leverage histatins for next-generation nanomedicine, enabling targeted delivery of antimicrobial and anticancer therapies at a molecular level. Their regenerative properties may inspire breakthroughs in tissue engineering and biomaterial development, fostering self-healing dental implants or bioactive wound dressings. Additionally, histatin-based gene-editing tools could enhance precision medicine, modulating cellular responses to fight resistant infections. By harnessing these extraordinary peptides, development of more personalised therapies for cancer, infectious diseases, and other medical challenges can be advanced.

## Figures and Tables

**Table 1:** List of studies describing histatins as the biomarkers of different diseases/processes.

Histatin Type	Disease Name	References
Histatin-1	Type I diabetes	[110]
Histatin-1	Periodontal disease	[111]
Histatin-1	AIDS	[112,113]
Histatin-5	Caries	[114,115]
Histatin-3 or Histatin-5	Alzheimer’s disease	[116]
Histatin-5	Stress	[117]
Histatin-2	Addictions including cocaine	[118,119]
Histatin-3	Obesity in pregnancy with periodontitis	[120]
Histatin-1	Bone diseases	[121]
Histatin-1	Aqueous-deficient dry eye disease (ADDE)	[122]
Histatin-3	Oral squamous cell carcinoma	[109]

**Table 2:** List of modified salivary histatins.

Name	Modification/ Engineering	Purpose/ Applications	References
Repeat-histatin-3 Repeat-histatin-3-repeat	Functional domain was repeated in tandem	5 times increased candidacidal activity	[148]
DR9-RR14	Hybrid of histatin-3 with statherin	Inhibit enamel demineralisation	[149]
Three histatin-5 proline variants 1:H21P 2:H19P/H21P 3:E16P/H19P/H21P	One or more residues were replaced with proline (potent α-helix breaker)	α-helix may not be important for candidacidal activity of histatin-5	[37]
ATCUN-C16 (modified histatin-5)	Contains two metal-binding centres, ATCUN motif (Cu-binding) and a Zn-binding motif	Assumes a more stable conformation and possesses nuclease activity, making it a suitable candidate for anticancer treatment and a biotechnological tool	[12]
Dhvar2 and modified dhvar2 (L7F) (modified histatin-5)	L7F (KRLFKEFLFSLRKY), required to facilitate peptide self-assembly into ordered nanostructures	Antimicrobial peptides with the ability to self-assemble into ordered amyloid-like nanostructures, facilitating their antibacterial activity and stable antifungal activities	[150]
P-113 Histatin-5 (C-terminal modification)	12-amino-acid sequence amidated on C terminus, reducing propensity to make an α-helix	Two-fold increase in fungicidal activity after amidation. LD50 = 2.3 ± 0.65 µg/mL	[49]
Histatin-5 (K17R)	Lysine at position 17 substituted for arginine in histatin-5	Confers increased resistance to proteolysis by Saps	[151]
Histatin-5 (K17L)	Lysine at position 17 substituted for leucine in histatin-5	Enhanced antifungal activity	[151]
Histatin-5 (K11R)	Lysine at position 11 substituted for arginine	Enhanced antifungal activity	[151]
W-histatin-5	Tryptophan (W) added in histatin-5 sequence	Prolonged fungicidal activity	[145]
Patents of histatin-5 and derivatives			
Cyclic analogues of histatin-5 U.S. Patent. 2011 November 10 (US 2014/0065119A1)	The invention focuses on the use of cyclic analogues of histatin-5 for the treatment of wounds. Cyclable amino acids can be incorporated to induce cyclisation in histatin-5 and its derivatives.	Cyclisation improves stability and cellular uptake of histatin-5. Therapeutically effective doses range from 0.01 mg to 100 mg per kg of body weight. A suitable absorbent hydrogel can be developed for topical application. Histatin-5, along with other therapeutic agents, can be used for wound healing.	[152,153]
WO 2016/060916 A1	The invention focuses on the utilisation of combined histatin-5 and histatin-1 as therapeutic agents for ocular surface diseases such as dry eyes.	Histatin-5, being a modulator of inflammatory cytokines, can be incorporated in anti-inflammatory formulations along with other therapeutics. The preferred weight-to-weight ratios of histatin-5 to cHistatin-1 were 1:1, 6:1, 1:10, and 1:15. Histatin-5 and histatin-1 were combined in ranges from 1 μg to 10 mg/mL. Both histatins were mixed with 0.1% to 1% glycerin to form sterile eye drops. Histatin-5, along with rapamycin, can be administered to treat dry eyes in patients suffering from autoimmune diseases such as Sjogren’s syndrome.	[152,154]
US 7781531 B2	Dentures conventionally made from poly (methyl methacrylate) lead to denture-induced stomatitis in the user due to adhesion of *C. albicans.*	This invention focuses on the incorporation of histatin-5 with phosphate-containing co-polymers in dentures. Phosphate anions facilitate the adhesion of cationic histatin molecule overdentures to limit the induced complications. Adsorption of histatin-5 increases with an increase in the negative charge on the polymer.	[152,155]
WO 2009/005798 A2	The invention is a histatin-5 derivative-based mouth rinse formulation with improved antifungal activity.	Amidation at the carboxyl terminus of the histatin-5 derivative resulted in a two-fold increase in antimicrobial activity.	[152,156]
US 2010/0202983 A1	The invention describes the utilisation of carrier agents for the delivery of histatins and their derivatives for the treatment of periodontal disease.	Carrier agents and histatins are covalently coupled to form a complex. The formed complex ensures sustained release of histatins with better penetration and retention.	[152,157]

**Table 3:** Modified histatins with side effects.

Name	Modification	Application	Reference
M21 (modified histatin-5)	K13T	Reduced fungicidal activity	[22]
M71 (modified histatin-5)	K13E	Reduced fungicidal activity	[22]
Dhvar2 (modified histatin-5)	Increased HIV-1 replication by promoting the envelope-mediated cell entry process	Modification of antimicrobial peptides in order to improve their activity against a pathogen may have unpredictable and unwanted side effects on other pathogens	[158]
LL37 and melittin (modified histatin-5)	Enhanced antifungal activity with increased growth of Lactobacillus species	Unwanted side effects on other commensals	[147]
Histatin-5— Histatin-5; Histatin-5—C16, C16—C16)	More potent histatin-5 molecules may be achieved by duplication of the functional domain of histatin-5 (C16, residues 9–24 of histatin-5)	Decreased candidacidal activity	[159]
